# CRISPR/Cas9-targeted mutagenesis of *Os8N3* in rice to confer resistance to *Xanthomonas oryzae* pv. *oryzae*

**DOI:** 10.1186/s12284-019-0325-7

**Published:** 2019-08-24

**Authors:** Young-Ah Kim, Hyeran Moon, Chang-Jin Park

**Affiliations:** 1 Department of Plant Biotechnology, Sejong University, Seoul, 05006 South Korea; 2 Department of Molecular Biology, Sejong University, Seoul, 05006 South Korea; 3 Plant Engineering Research Institute, Sejong University, Seoul, 05006 South Korea

**Keywords:** CRISPR/Cas9, Disease resistance, *Os8N3*, Rice, *xa13*, *Xanthomonas oryzae* pv. *oryzae*

## Abstract

**Background:**

Genome editing tools are important for functional genomics research and biotechnology applications. Recently, the clustered regularly interspaced short palindromic repeats (CRISPR)/CRISPR-associated protein-9 (Cas9) system for gene knockout has emerged as the most effective genome-editing tool. It has previously been reported that, in rice plants, knockdown of the *Os8N3* gene resulted in enhanced resistance to *Xanthomonas oryzae* pv. *oryzae* (*Xoo*), while displaying abnormal pollen development.

**Results:**

The CRISPR/Cas9 system was employed to knockout rice *Os8N3*, in order to confer enhanced resistance to *Xoo*. Analysis of the genotypes and edited *Os8N3* in T_0_, T_1_, T_2_, and T_3_ transgenic rice plants showed that the mutations were transmitted to subsequent generations, and homozygous mutants displayed significantly enhanced resistance to *Xoo*. Stable transmission of CRISPR/Cas9-mediated *Os8N3* gene editing without the transferred DNA (T-DNA) was confirmed by segregation in the T_1_ generation. With respect to many investigated agronomic traits including pollen development, there was no significant difference between homozygous mutants and non-transgenic control plants under greenhouse growth conditions.

**Conclusion:**

Data from this study indicate that the CRISPR/Cas9-mediated *Os8N3* edition can be successfully employed for non-transgenic crop improvements.

**Electronic supplementary material:**

The online version of this article (10.1186/s12284-019-0325-7) contains supplementary material, which is available to authorized users.

## Background

Rice (*Oryza sativa* L.) is one of the most important cereal crops in the world, directly feeding more people than any other crop. Bacterial blight, caused by *Xanthomonas oryzae* pv. *oryzae* (*Xoo*), is a prevalent and destructive rice disease that causes serious production loss worldwide (Zhang and Wang 2013). Enhancing rice plants’ resistance to *Xoo* is known to be an economical and effective approach for managing rice bacterial blight.

*Xoo* pathogenicity depends on a specific class of virulence factors, called transcription activator-like (TAL) effectors, which mimic plant transcriptional activators (Hutin et al. 2015; Blanvillain-Baufume et al. 2017). The TAL effectors target the host nucleus, where they bind to specific promoter elements of the plant genes and activate their expression, reprogramming the plant transcriptome (Schornack et al. 2013). The genomes of *Xanthomonas* strains typically contain highly variable numbers of TAL effectors between Asian *Xoo* (15–26), African *Xoo* (8–10), and North-American *Xoo* (0) (Erkes et al. 2017). The rice genes targeted by TAL effectors have been identified as host disease-susceptibility genes, acting as major susceptibility factors during rice and *Xoo* interactions. In some cases, DNA polymorphisms in the so-called TAL effector binding elements (EBEs), located at the promoter region of the susceptibility gene, lead to no development of the disease (Yang et al. 2006; Hutin et al. 2015). Rice *Os8N3* (also known as *OsSWEET11*), which belongs to the Sugar Will Eventually be Exported Transporters (SWEET) family of sugar transporters, represents one of the susceptibility genes induced by TAL effectors (Yang et al. 2006; Chen 2014). The expression of *Os8N3* is induced by strains of *Xoo* carrying *pthXo1*, which encodes the TAL effector PthXo1 (Yang et al. 2006; Yuan et al. 2009). PthXo1 from *Xoo* strain PXO99 directly activates *Os8N3* through recognition of TAL EBEs located at the promoter region of *Os8N3* (Romer et al. 2010). The recessive resistance gene *xa13* occurs as a series of natural alleles of the susceptibility gene *Os8N3* (Yang et al. 2006; Yuan et al. 2009). Although it has not been clearly demonstrated, Os8N3 is believed to remove toxic copper from xylem vessels where *Xoo* multiplies and spreads (Yuan et al. 2010), and make nutrients easily available to *Xoo* for its growth and virulence to cause disease (Chen et al. 2010; Chen et al. 2012).

Genome editing technologies enable precise modification of DNA sequences in vivo and promise a novel revolution in crop improvement (Sun et al. 2016; Feng et al. 2013). The clustered regularly interspaced short palindromic repeats (CRISPR)/CRISPR-associated protein-9 (Cas9) system has revolutionized genome editing and become widely popular because of its specificity, simplicity, and versatility. It allows targeted genome editing in organisms guided by a customizable small noncoding RNA called single guide RNA (sgRNA). Once susceptibility genes targeted by TAL effectors have been identified, the CRISPR/Cas9-mediated genome editing strategy can be employed to create a target mutation in the susceptibility genes. Although it was not edited by the CRISPR/Cas9, *Os11N3* (also known as *OsSWEET14*), the susceptibility gene targeted by AvrXa7 and PthXo3, has been edited by Transcription Activator-Like Effector Nucleases (TALENs) to create bacterial blight-resistant rice through disrupting the EBE site in the promoter region (Li et al. 2012; Blanvillain-Baufume et al. 2017). It can also be applied to negative regulators of disease resistance that have been studied for the last decades (Grand et al. 2012; Wang et al. 2015; Chern et al. 2005). However, to date, only a few examples of improvement of disease resistance using the CRISPR/Cas9 approach have been reported (Wang et al. 2016; Pyott et al. 2016; Peng et al. 2017). For *Os8N3*, studies on its knockdown rice plants using the gene silencing system and promoter mutations reported that they showed enhanced resistance to *Xoo* while displaying abnormal pollen development (Yang et al. 2006; Chu et al. 2006). Recently, CRISPR/Cas9-mediated knockout of *Os8N3* displayed decreased sucrose concentration in the embryo sacs and defective grain filling, suggesting that *Os8N3* plays important role in sucrose transport during early stage of rice grain filling (Ma et al. 2017; Yang et al. 2018).

Here, the CRISPR/Cas9-target mutagenesis of *Os8N3* in Kitaake, a Japonica rice cultivar, is reported. The homozygous mutant lines carrying edited *Os8N3* displayed significantly enhanced resistance to *Xoo* with normal pollen development. It was possible to select resistant mutant lines not containing the transferred DNA (T-DNA) by segregation in the T_1_ generation.

## Results

### *Os8N3* in the rice cultivar Kitaake

**Fig. 1:**
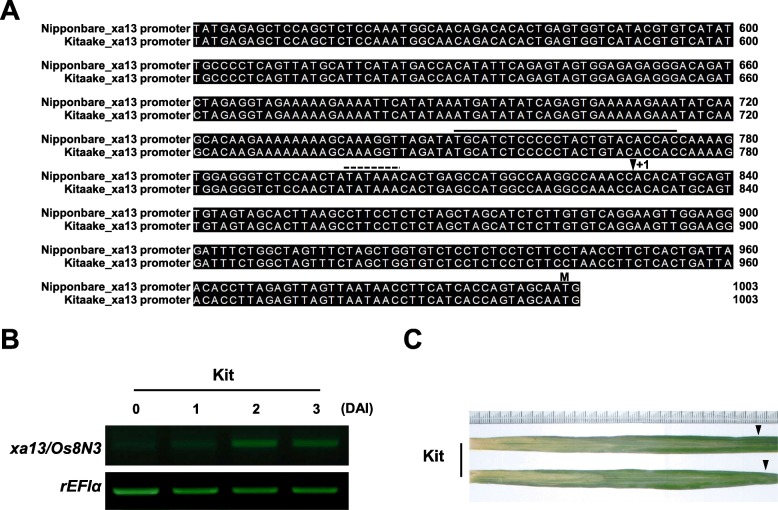
*Os8N3* is a susceptibility gene for *Xoo* strain PXO99 in rice cultivar Kitaake. **a** Promoters containing a PthXo1 EBE (upper line) from Nipponbare and Kitaake displayed 100% identity to each other. The putative TATA box is shown by a dashed line. The transcription start site is represented by a vertical arrowhead noted as + 1. The translational initiating ATG codon is shown as ‘M’. **b** Expression of *Os8N3* is elevated after inoculation with *Xoo* strain PXO99 in Kitaake. Rice *elongation factor 1α* (*rEF1α*) was used as an internal control. **c** Kitaake exhibited a susceptible phenotype with long water-soaked lesions after inoculation with PXO99. The lesions were photographed 12 days after inoculation (DAI) and arrowheads indicated the end of the lesion

*Os8N3* was originally isolated as a susceptibility gene from the rice cultivar Nipponbare (Yang et al. 2006) and later, the EBE in its promoter element bound and activated by TAL effector PthXo1 of PXO99 was determined experimentally (Romer et al. 2010). In this study, rice cultivar Kitaake was investigated to see if it also carries the EBE sequence in the *Os8N3* promoter region. Using the Kitaake database (Li et al. 2017), the promoter sequence of the *Os8N3* gene, ranging from − 1000 bp to − 1 bp relative to the ATG start codon, was analyzed (Fig. 1a). The putative TATA box (TATAAA) is located at − 32 upstream of the transcription start site (+ 1). The promoter region including PthXo1 EBE (TGCATCTCCCCCTACTGTACACCAC), ranging from − 80 bp to − 56 bp upstream of the transcription start site, displayed 100% identity to Nipponbare (Yang et al. 2006). After inoculation with strain PXO99, Kitaake displayed strong induction of *Os8N3* two days after inoculation (DAI) (Fig. 1b) and long water-soaked lesions (approximately 13–14 cm) 12 DAI (Fig. 1c). These results suggest that Kitaake carries a functional susceptible gene *Os8N3*, whose expression is induced by PXO99 possessing the TAL effector PthXo1.

### CRISPR/Cas9 design for *xa13/Os8N3* editing

In monocot plants, the rice U3 small nuclear RNA promoter (OsU3) is generally used to express sgRNA (Belhaj et al. 2013). Recently, the efficiency of mutations targeted by sgRNAs driven by different small nuclear RNA promoters including OsU3, OsU6a, OsU6b, and OsU6c, were compared in an Indica cultivar 93–11 (Ma et al. 2015b). OsU6a was slightly more efficient in driving genome editing than the other promoters. It has also been reported that U6 promoters derived from the target plants function better than heterologous U6 promoters (Sun et al. 2015). Therefore, it was decided to use the OsU6a promoter isolated from the Japonica cultivar Kitaake. The OsU6a promoter amplified from Kitaake contains five single-nucleotide substitutions and one 5-bp deletion compared with one from Indica cultivar 93–11 (Additional file 1: Figure S1). The Arabidopsis U6 promoter in the CRISPR/Cas9 vector, pHAtC (Kim et al. 2016), was replaced with the Kitaake OsU6a promoter, and the resulting OsU6a::pHAtC was used for rice CRISPR/Cas9-mediated target mutagenesis.

**Fig. 2:**
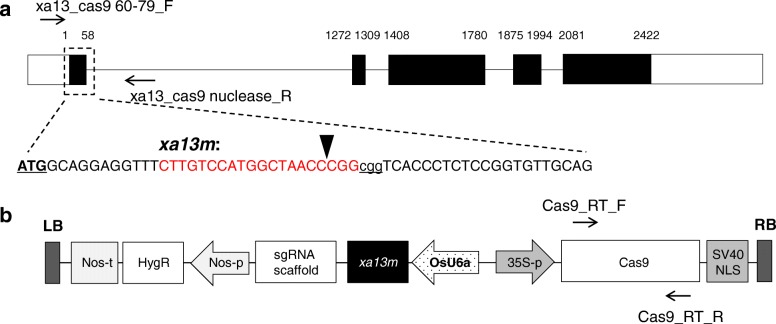
Schematic representation of CRISPR/Cas9-mediated targeted mutagenesis in the rice *Os8N3* gene. **a** Schematic diagram of *Os8N3* gene and *xa13m* targeting sequence*.* Rice *Os8N3* contains five exons, represented by black rectangles, and the untranslated region portion, represented by white rectangles. The enlarged area indicated by the black broken line shows the coding sequence and position of the first exon of *Os8N3*. The 20-bp sgRNA targeting sequence (*xa13m*) and protospacer adjacent motif (PAM) sequence are shown in red and in underlined lower-case letters, respectively. The vertical arrowhead indicates an expected cleavage site. The underlined bold ATG indicates a translation initiation codon. **b** T-DNA region of the recombinant OsU6a::*xa13m-sgRNA*/pHAtC vector carrying *xa13m-sgRNA* under the control of the OsU6a promoter. Expression of Cas9 is driven by the Cauliflower mosaic virus 35S (CaMV35S) promoter; expression of the *xa13m-sgRNA* is driven by the OsU6a promoter; expression of *hygromycin* (HPT) is driven by the nopalin synthase (NOS) promoter; NLS: nuclear localization signal of Simian virus 40 (SV40) large T antigen; nos-t: gene terminator; LB and RB: left and right border, respectively. Primers used in the PCR are indicated by black arrows

To design a CRISPR/Cas9 that targets the *Os8N3* gene, a 20-bp nucleotide sequence (*xa13m*) in the first exon of *Os8N3* was chosen as the target site (Fig. 2a). The *xa13m* targeting sequence and protospacer adjacent motif (PAM) sequence are represented in red and in underlined lower-case letters, respectively. The predicted Cas9 cleavage site (vertical arrowhead) in the coding region of the gene was 31 bp downstream from the ATG initiation codon. The recombinant binary plasmid, OsU6a::*xa13m-sgRNA*/pHAtC, carrying *xa13m-sgRNA* targeting the *Os8N3* gene under the control of the OsU6a promoter, was then constructed based on the OsU6a::pHAtC (Fig. 2b).

### CRISPR/Cas9-mediated targeted mutagenesis of *xa13/Os8N3*

**Fig. 3:**
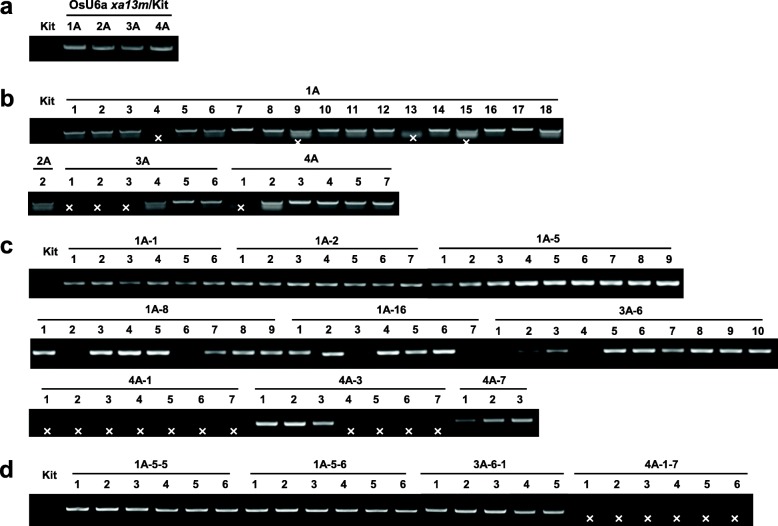
Generation of transgenic rice plants carrying the Cas9 transgene with a sgRNA targeting the *Os8N3* gene. Genotyping was performed using the specific primers for Cas9, Cas9_RT_F and Cas9_RT_R (see Fig. 2b), from four independently transformed plants and their progenies (OsU6a *xa13m*/Kit T_0_, T_1_, T_2_, and T_3_ generations). Genomic DNAs were extracted from Kit (Kitaake) and OsU6a *xa13m*/Kit T_0_ (**a**), T_1_ (**b**), T_2_ (**c**), and T_3_ (**d**). ‘ × ’ indicates PCR negative

After Kitaake was transformed with OsU6a::*xa13m-sgRNA*/pHAtC using Agrobacterium-mediated transformation, four independent transgenic Kitaake plants (OsU6a *xa13m*/Kit T_0_, 1A, 2A, 3A, and 4A) were generated. The putative transgenic plants were subjected to polymerase chain reaction (PCR)-based selection using the Cas9-specific primers, Cas9_RT_F and Cas9_RT-R (Fig. 2b), and all of them generated a Cas9-specific 400-bp amplicon (Fig. 3a). To further investigate CRISPR/Cas9-targeted mutagenesis of *Os8N3*, the target-containing amplicons obtained from all PCR-positive transgenic plants were directly sequenced and analyzed by decoding via the Degenerate Sequence Decoding method (Liu et al. 2015; Ma et al. 2015a). Rice plants are diploid with two copies of each gene, one copy on each chromosome of a chromosome pair. Therefore, when CRISPR/Cas9 is inserted into the genome and begins to function, one or both copies of the target gene *Os8N3* can be cleaved and mutated, generating five possible genotypes in the transgenic plants: homozygote, biallele, heterozygote, chimera, and wild type (WT). In four T_0_ transgenic plants, there was only one homozygous mutation, 1-bp insertion (+A), in 4A, whereas no target sequence changes could be detected in the other plants (T_0_ in Table 1 and Additional file 2: Figure S2).

**Table 1:**
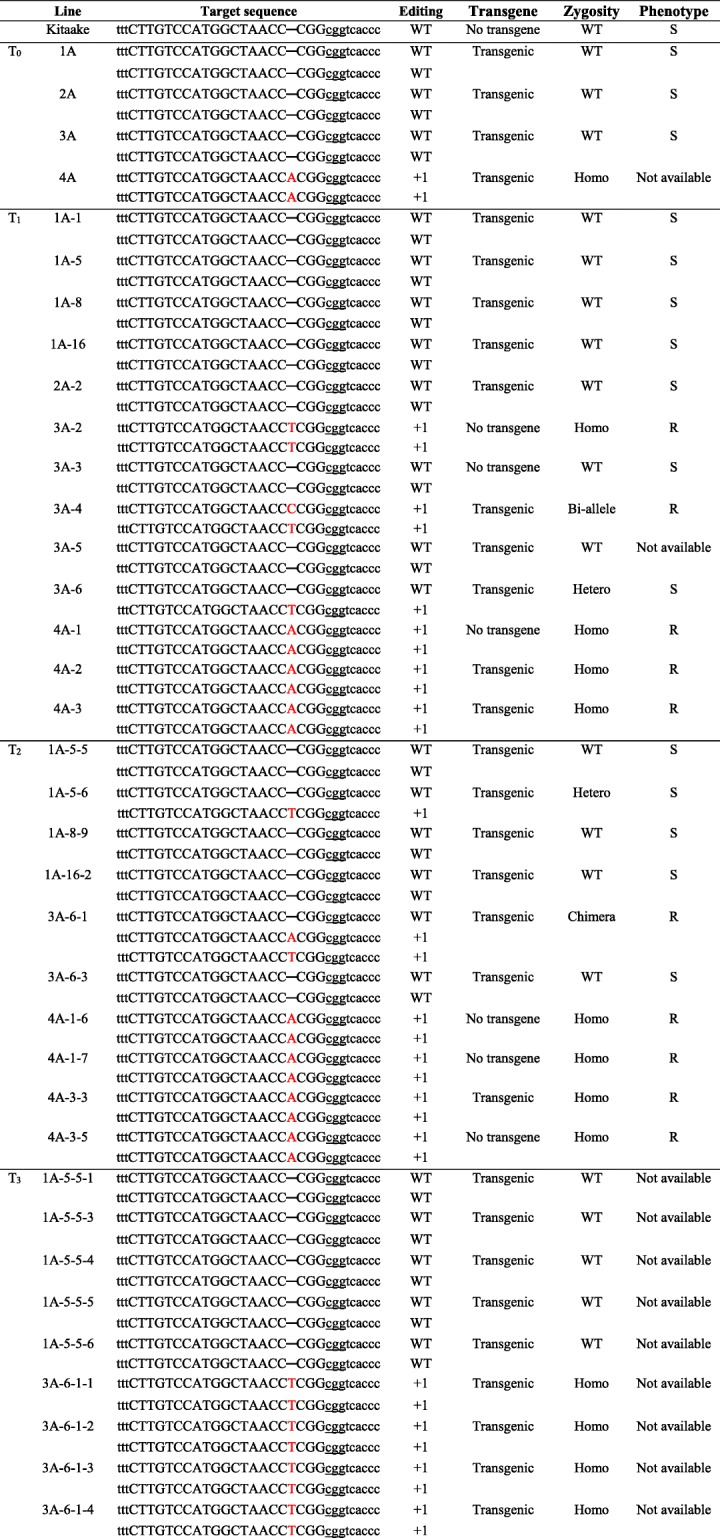
Transmission and segregation of CRISPR/Cas9-mediated target mutagenesis from T_0_, T_1_, T_2_, and T_3_ of the OsU6a *xa13m*/Kit transgenic plant. The recovered mutated alleles of the *xa13/Os8N3* gene in the OsU6a *xa13m*/Kit transgenic plant are shown below the Kitaake sequence. Nucleotide sequences at the target sites are shown in black capital letters and black dashes. PAM motifs are underlined. Red capital letters indicate the inserted nucleotide. The genotype of the mutation is indicated at the right of each sequence. WT indicates the nucleotide sequences identical to the *Os8N3* gene in Kitaake plants. “+” indicates the insertion of the indicated number of nucleotides. No transgene: PCR negative for *Cas9* gene; Transgenic: PCR positive for *Cas9* gene; S: susceptible to PXO99; R: resistant to PXO99; Not available: inoculation data are not available

### Inheritance of *Os8N3* mutations and enhanced resistance to *Xoo*

To determine if and how the CRISPR/Cas9-targeted mutagenesis of *Os8N3* by OsU6a::*xa13m-sgRNA*/pHAtC was transmitted to the next generation, all OsU6a *xa13m*/Kit T_0_ transgenic plants were self-pollinated and the targeted *Os8N3* of some T_1_ transgenic plants was directly sequenced and analyzed (Fig. 3b, Table 1, and Additional file 3: Figure S3). The homozygous mutated T_0_ line (4A) produced homozygous mutated T_1_ progeny (4A-1, 4A-2, and 4A-3) and did not display additional different mutations. There was no mutation observed in the sequenced T_1_ progenies of the WT 1A, and 2A lines. However, new targeted sequence changes were detected in the T_1_ progeny of the WT 3A line. Previously, sequencing results indicated a putative WT genotype of the targeted *Os8N3* in the T_0_ 3A line, whereas three (3A-2, 3A-4, and 3A-6) out of the five sequenced T_1_ progenies of the WT 3A line displayed a 1-bp insertion (Table 1): 3A-2 was homozygous; 3A-4 was bi-allelic; and 3A-6 was heterozygous.

**Fig. 4:**
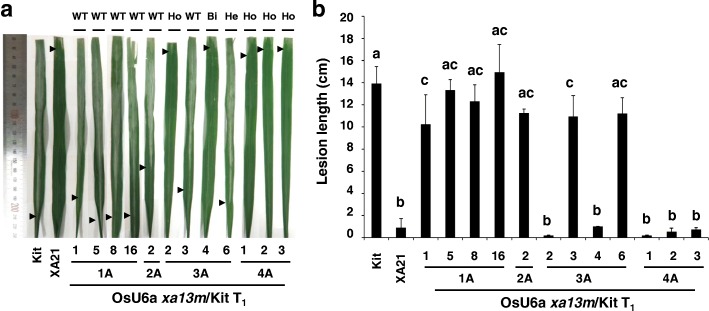
CRISPR/Cas9-mediated mutagenesis in both *Os8N3* alleles conferred enhanced resistance to *Xoo*. **a** Bacterial blight resistance phenotypes of the *xa13* mutant rice lines (T_1_). Rice plants 12 DAI with *Xoo*. From left to right: Kitaake (Kit), transgenic line (XA21, 7A-8) carrying *Xa21* driven by the *ubiquitin* promoter, and transgenic lines (OsU6a *xa13m*/Kit, T_1_) carrying the OsU6a::*xa13m-sgRNA*/pHAtC construct. Arrowheads indicated the end of the lesion. WT; wild type: Ho; homozygous: Bi; bi-allelic: He; Heterozygous. **b** Lesion lengths measured 12 DAI in Kitaake, XA21, and OsU6a *xa13m*/Kit T_1_. Error bars in the graph represent standard error of at least three leaves from each plant. Letters indicate a significant difference at *P* < 0.050 by Tukey’s HSD test

To characterize the bacterial blight resistance phenotype of the mutant lines, T_1_ lines (progeny of OsU6a *xa13m*/Kit 1A, 2A, 3A, and 4A) with different types of allelic mutations were inoculated with PXO99 at the eight-week stage (Fig. 4a). Kitaake and transgenic Kitaake carrying *Xa21* (XA21), driven by the *ubiquitin* promoter, were used as the susceptible and resistant control for PXO99, respectively (Park et al. 2010). As expected, while the XA21 plant was highly resistant, displaying short lesions, the inoculated leaves of the Kitaake plants developed long water-soaked lesions typical of bacterial blight disease. Homozygous (OsU6a *xa13m*/Kit 3A-2, 4A-1, 4A-2, and 4A-3) and bi-allelic (3A-4) *xa13* mutant plants displayed a robust resistance phenotype compared with heterozygous (3A-6) mutant and Kitaake control plants (T_1_ in Table 1 and Fig. 4a). The differences were further evaluated by quantification of the lesion lengths and significance analysis using Tukey’s HSD test (Fig. 4b). Homozygous and bi-allelic mutant plants displaying a resistance phenotype showed no significant differences in lesion lengths compared with the XA21 plants. These results indicated that the homozygous and bi-allelic mutant lines were significantly different from Kitaake and heterozygous mutant plants, and that CRISPR/Cas9-mediated mutagenesis in both *Os8N3* alleles conferred robust resistance to PXO99.

**Fig. 5:**
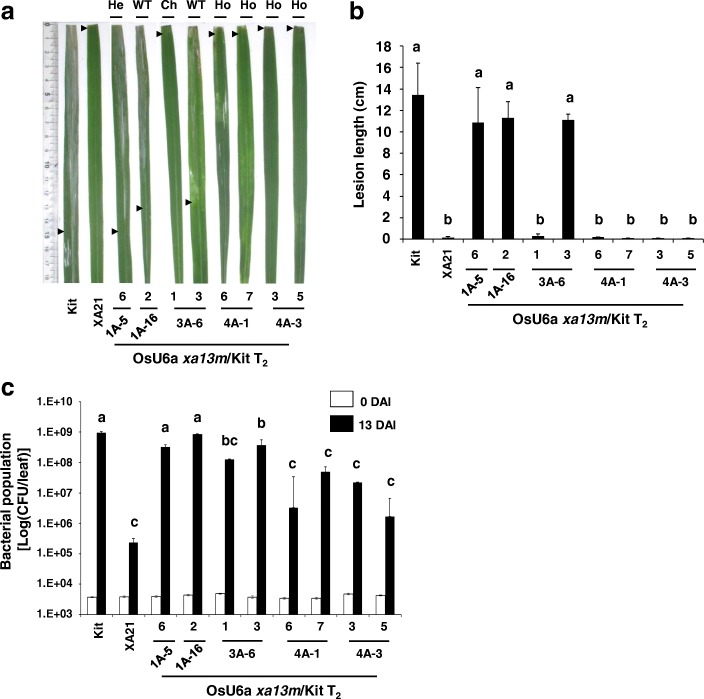
Homozygous mutants in both *Os8N3* alleles displayed enhanced resistance to *Xoo*. Transgenic Kitaake plants targeting *xa13* (OsU6a *xa13m*/Kit T_2_) display enhanced resistance to *Xoo*. **a** Inoculation results for mutant rice lines 12 DAI with *Xoo*. From left to right: Kitaake (Kit), transgenic line (XA21, 7A-8) carrying *Xa21* driven by the *ubiquitin* promoter, and transgenic lines (OsU6a *xa13m*/Kit, T_2_) carrying the OsU6a::*xa13m-sgRNA*/pHAtc construct. Arrowheads indicated the end of the lesion. He; Heterozygous; WT; wild type: Ch; chimeric: Ho; homozygous. **b** Lesion lengths measured 12 DAI in Kitaake, XA21, and OsU6a *xa13m*/Kit T_2_. Error bars in the graph represent standard error of at least three leaves from each plant. Letters indicate a significant difference at P < 0.050 by Tukey’s HSD test. **c** Bacterial population in Kitaake, XA21, and OsU6a *xa13m*/Kit T_2_ plants 0 and 12 DAI, determined by the number of CFU per inoculated leaf. Error bars represent standard deviation from at least three technical replicates. Letters indicate a significant difference at P < 0.050 by Tukey’s HSD test

To further investigate the inheritance of targeted mutations in later generations, the genotypes of several OsU6a *xa13m*/Kit T_2_ plants were analyzed and inoculated with PXO99. New allelic mutation was detected in the T_2_ progeny of WT 1A-5. Although all sequenced T_0_ and T_1_ generations of the 1A line carry WT *Os8N3*, T_2_ progeny (1A-5-6) of the 1A line displayed a heterozygous 1-bp insertion (+T) mutation (Table 1 and Additional file 4: Figure S4). Heterozygous mutated 3A-6 (+T) produced chimera 3A-6-1 with three distinct alleles detected at the target site, displaying additional different mutations (+A). All T_1_ plants derived from the homozygous T_0_ mutant plant (4A) and T_2_ plants derived from homozygous T_1_ mutant plants (4A-1 and 4A-3) were homozygous for the same mutations (Table 1). All homozygous mutant lines (4A-1-6, 4A-1-7, 4A-3-3, and 4A-3-5) and chimera (3A-6-1) displayed significantly short lesion lengths (Fig. 5a and b) and low bacterial populations compared with the heterozygous mutant (1A-5–6) and Kitaake plants (Fig. 5c). These results indicate that the mutations in these homozygous mutant lines and enhanced resistance to PXO99 were stably transmitted to the next generation.

### Main agronomic traits in *xa13* mutants

To determine whether mutations in the *Os8N3* gene affect agronomic traits, two independent homozygous mutant lines (T_3_) were analyzed by measuring their plant height, flag leaf length/width, the number of productive panicles, and panicle length (Table 2, Additional file 5: Figure S5 and Additional file 6: Figure S6). Tukey’s HSD test indicated that the mutant lines displayed no significant difference to Kitaake, in terms of the investigated agronomic traits, under our greenhouse conditions.

**Table 2:** Analysis of the agronomic traits of T_3_ mutant lines

	Plant height (cm)	Flag leaf length (cm)	Flag leaf width (mm)	No. of productive panicles	Panicle length (cm)
Kitaake	69.8 ± 4.1^a^	27.8 ± 4.9^a^	11.7 ± 0.9^a^	3.0 ± 0.0^a^	11.1 ± 2.1^a^
Progeny of 3A-6-1	65.6 ± 6.4^a^	26.9 ± 4.0^a^	12.7 ± 0.5^a^	3.0 ± 0.0^a^	12.1 ± 1.4^a^
Progeny of 4A-1-7	65.7 ± 7.9^a^	29.1 ± 4.8^a^	12.0 ± 0.4^a^	2.5 ± 1.1^a^	12.0 ± 2.2^a^

The results shown are from more than three homozygous mutants of each mutant line, and are represented as the mean ± SE. The values marked with the same letter (^a^) are non-significantly different (*P* < 0.050, Tukey’s HSD test)

**Fig. 6:**
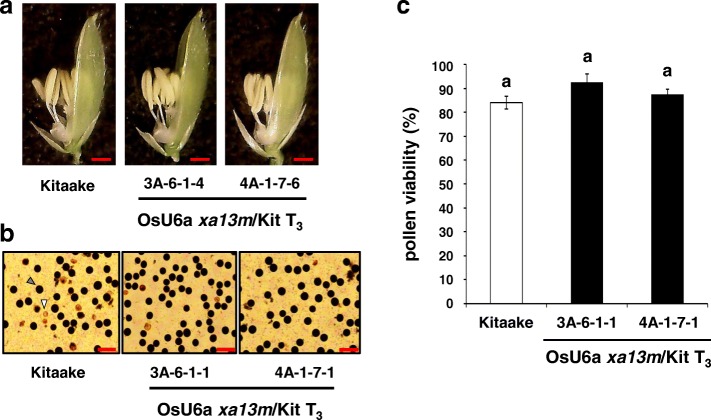
Pollen viability of the homozygous *xa13* mutants. **a** Anthers in mature spikelets of Kitaake, homozygous mutant (T_3_, 3A-6-1-4), and homozygous mutant (T_3_, 4A-1-7-6). Scale bars, 1 mm. **b** Representative images of pollen viability tests from Kitaake and homozygous mutants (T_3_, 3A-6-1-1 and 4A-1-7-1). Viable pollen grains are stained dark (gray arrow) and sterile pollen grains are stained light yellow (white arrow). Scale bars, 100 μm. **c** Statistical analysis of pollen viability of Kitaake, homozygous mutants (T_3_, 3A-6-1-1 and 4A-1-7-1) lines. Pollen viability percentage was calculated relative to the total pollen counted in three microscopic images

Previously, *Os8N3* knockdown transgenic plants displayed abnormal pollen development (Yang et al. 2006; Chu et al. 2006). To investigate whether *Os8N3* knockout mutations affect pollen development, their pollen developments were assessed (Fig. 6). The phenotypical analysis showed that two independent homozygous T_3_ mutant lines (3A-6-1-4 and 4A-1-7-6) exhibited normal golden yellow anthers (Fig. 6a). In addition, pollen grains from Kitaake and two independent homozygous T_3_ mutant lines (3A-6-1-1 and 4A-1-7-1) were stained with iodine potassium iodide (I_2_-KI) (Fig. 6b). Dark-stained pollen grains (black in color) were considered viable and those that were lightly stained (yellow in color) were considered sterile. Homozygous mutants (3A-6-1-1 and 4A-1-7-1) displayed similar pollen viabilities to Kitaake, under our greenhouse conditions (Fig. 6c). The seed-setting rates and grain fillings were further analyzed in the *Os8N3* knockout mutant lines (Additional file 7: Figure S7). Although, under greenhouse conditions, the caryopses from two independent homozygous mutants (3A-6-1-1 and 4A-1-7-1) were slightly wrinkled as they matured (Additional file 7: Figure S7c), no significant alteration in the seed-setting rate was observed between progeny of two homozygous mutants (3A-6-1 and 4A-1-7) and Kitaake plants (Additional file 7: Figure S7a and S7b).

### Selection of transgene-free mutant rice lines

To select rice plants harboring the mutation in *Os8N3* but without the T-DNA of the OsU6a::*xa13m-sgRNA*/pHAtC construct, PCR and phenotypic analysis for the OsU6a *xa13m*/Kit T_0_, T_1_, and T_2_ plants was performed. Thirty-one segregating T_1_ plants were analyzed and six of them (19.35%) did not generate a Cas9-specific amplicon from the T-DNA (Fig. 3b). Similarly, PCR analysis also failed to detect the T-DNA in 11 out of the 65 segregating T_2_ plants (16.92%) derived from nine T_1_ plants (1A-1, 1A-2, 1A-5, 1A-8, 1A-16, 3A-6, 4A-1, 4A-3, and 4A-7) (Fig. 3c). Notably, the 4A-1 plant was a Cas9-free homozygous mutant harboring the desired *xa13/Os8N3* modifications (Fig. 3b and Fig. 4, and Additional file 3: Figure S3 and Additional file 4: Figure S4). None of the seven T_2_ plants derived from the T_1_ mutant plant 4A-1 generated the Cas9-specific amplicon (Fig. 3c). Two (4A-1-6 and 4A-1-7) out of the seven carried a 1-bp insertion (+A) and displayed significantly enhanced resistance to PXO99 (Fig. 5), which has also been observed in their parent (4A-1) (Fig. 4). The T_3_ plant (4A-1-7-1) not generating the Cas9-specific amplicon carried the same *Os8N3* modification observed in the T_2_ mutant plant 4A-1-7 (Fig. 3d and Additional file 4: Figure S4 and Additional file 5: Figure S5). These results indicate that T-DNA-free mutant plants carrying the desired gene modifications can be acquired through genetic segregation in T_1_, T_2_, and T_3_ generations.

## Discussion

The CRISPR/Cas9 system has been widely used to provide new avenues in crop improvements in rice, tomato, wheat, and maize (Xu et al. 2015; Feng et al. 2013; Wang et al. 2016; Ito et al. 2015; Wang et al. 2014; Zhou et al. 2014). In this study, OsU6a::pHAtC, which replaced the Arabidopsis U6 promoter in the pHAtC vector (Kim et al. 2016) with the OsU6a promoter of Kitaake, was constructed for rice CRISPR/Cas9-mediated target mutagenesis. Using the OsU6a::pHAtC, targeted mutagenesis in the recessive resistance gene, *Os8N3*, was generated*.*

One *xa13* mutant line 4A (T_0_) from four independent transgenic OsU6a *xa13m*/Kit plants carrying OsU6a::*xa13m-sgRNA*/pHAtC was obtained. However, new targeted sequence changes were continuously detected in the transgenic OsU6a *xa13m*/Kit plants in subsequent generations. For example, two additional independent mutant lines (progenies of 3A and 1A-5) were identified in the T_1_ and T_2_ generations, respectively. Except for line 2A, which was lost in T_1_, all available lines in T_2_ were successfully mutated at the target sequence. Because the CRISPR/Cas9 system has been shown to be active in heterozygous and chimeric plants (Xu et al. 2015; Zhou et al. 2014), it is possible for the WT allele to be continuously modified in subsequent generations. Therefore, non-mutated transgenic plants, in which the OsU6a::*xa13m-sgRNA*/pHAtC construct remained active, continually cleaved the target site for generations, resulting in new mutations. Multiple mutations were also detected at the target site in the T_2_ mutant plant 3A-6-1. Because 3A-6 was heterozygous, the presence of a chimeric mutation may result from delayed cleavage in the primary embryogenic cell of 3A-6-1. This chimeric mutation by the CRISPR/Cas9 system is likely a common phenomenon and has been reported in many plant species including rice (Xu et al. 2015; Feng et al. 2013; Wang et al. 2016), Arabidopsis (Feng et al. 2014), and tomato (Ito et al. 2015).

Regarding all examined agronomic traits, there was no significant difference between T_3_ homozygous mutants and Kitaake plants under greenhouse growth conditions. The homozygous mutant plants had a similar height, flag leaf length and width, number of productive panicles, panicle length, and pollen viability to Kitaake plants. It has been previously reported that *Os8N3* is expressed at a high level in panicles and anthers during pollen development (Chu et al. 2006; Yang et al. 2006). Consistent with these observations, although detailed molecular mechanisms have not been elucidated, *Os8N3*-silenced rice plants displayed reduced fertility, and most pollen grains were defective (Chu et al. 2006; Yang et al. 2006). Therefore, *Os8N3*, conferring disease resistance by expressional loss-of-function in rice, has been considered an essential constituent for pollen development. However, in this study, homozygous mutants in both *Os8N3* alleles were generated, and the mutations were stably transmitted to later generations, T_3_. The homozygous T_3_ mutant plants had normal pollen development, and most pollen grains were well preserved, in comparison with ones from Kitaake plants.

Thus far, it has been believed that Os8N3 plays roles in both copper and sugar transport, indicating its complex function in copper/sugar metabolism and signaling (Chen et al. 2010; Chen 2014; Yuan et al. 2010). However, no one dissected the molecular connection between *Xoo* resistance by copper/sugar metabolism and pollen development. Among the different in vivo functions of xa13/Os8N3, knockout mutation, in particular, displayed enhanced resistance against *Xoo* without affecting pollen development. It is not yet understood why OsU6a *xa13m*/Kit mutant lines did not display the sterile phenotype previously observed in *Os8N3*-knockdown rice plants (Chu et al. 2006; Yang et al. 2006). Because frameshift mutations of *Os8N3* in OsU6a *xa13m*/Kit lines are located at the very beginning of the Os8N3 polypeptide, it is very unlikely that the mutated polypeptide is functional. Lack of a functional Os8N3 protein in the mutant lines was also supported by a robust resistant phenotype of the homozygous mutant lines, but not heterozygous or Kitaake plants. Therefore, it is possible that there is a novel gene genetically compensating essential pollen development directly or indirectly in homozygous OsU6a *xa13m*/Kit mutant lines. Genetic compensation was recently proposed to explain increasing numbers of studies revealing phenotypic differences between knockouts and knockdowns in plants (Gao et al. 2015; Braun et al. 2008; Chen et al. 2014) and animals (Young et al. 2009; De Souza et al. 2006; Daude et al. 2012; McJunkin et al. 2011; Law and Sargent 2014; Evers et al. 2016; Karakas et al. 2007; Morgens et al. 2016; Kok et al. 2015; Rossi et al. 2015). For example, similar to Os8N3, there have been studies on Arabidopsis auxin-binding protein 1 (ABP1) that revealed phenotypic differences between knockouts and knockdowns (Gao et al. 2015; Braun et al. 2008; Chen et al. 2014). Inducible *abp1* knockdown lines showed defects in shoot and root growth, cell remodeling, or clathrin-mediated endocytosis of PIN auxin efflux carriers (Braun et al. 2008; Paque et al. 2014; Robert et al. 2010). However, *abp1* knockout mutants generated by CRISPR/Cas9 are indistinguishable from wild type plants at every developmental stage analyzed (Gao et al. 2015). Although one possible explanation for the difference is off-target effects of *ABP1* antisense RNA, it is not yet understood how independent *abp1* knockdown lines, which generate fundamentally different approaches for functional down-regulation of the *ABP1* gene, display indistinguishable morphological defect phenotypes (Michalko et al. 2016). Recently, genetic compensation was studied in depth on zebrafish (Rossi et al. 2015). While knockdown of zebrafish *EGF-like domain 7* (*egfl7*), an endothelial extracellular matrix gene, leads to severe vascular defects, most *egfl7* mutants display no obvious defects (Rossi et al. 2015). *Elastin microfibril interfacer* (*Emilin*) genes were proposed as compensating genes in the *edgl7* knockout mutants (Rossi et al. 2015). Supporting this hypothesis, *Os8N3* mutants showed increased expressions of several *SWEET* genes such as *OsSWEET3a*, *OsSWEET6b*, *OsSWEET13*, and *OsSWEET15* (Ma et al. 2017; Yang et al. 2018) and double mutants of *Os8N3* and *OsSWEET15* displayed much more wrinkled grain morphology, compared with single *Os8N3* mutant (Yang et al. 2018). These reports suggest that some of *SWEET* genes are able to at least partially compensate for the lack of *Os8N3*. Currently, we are trying to identify candidate genes that compensate for *xa13/Os8N3* in the pollen development pathway without affecting *Xoo* resistance in homozygous mutant lines.

## Conclusions

In summary, the CRISPR/Cas9 system was highly efficient in generating *Os8N3* gene editing in rice. Mutant lines harboring the desired modification in *Os8N3* but without the T-DNA of the OsU6a::*xa13m-sgRNA*/pHAtC were obtained. T-DNA-free homozygous mutant lines displayed significantly enhanced resistance to *Xoo* and normal pollen development. This study provides a successful example of improving bacterial blast resistance using CRISPR/Cas9 technology.

## Materials and methods

### Plant and pathogen materials

Rice cultivar Kitaake (*Oryza sativa* L. ssp. *Japonica*) was generously provided by Prof. Pamela Ronald (University of California Davis, USA). Rice plants in this study were maintained in the greenhouse facility at Sejong University in Korea. *Xoo* strain PXO99 was used in this study. PXO99 was cultured in peptone sucrose agar media (PSA: peptone 10.0 g/L, sucrose 1.0 g/L, L-glutamic acid 1.0 g/L, and agar 16.0 g/L) containing 15.0 mg/L cephalexin at 28 °C for two days (Bai et al. 2000).

### Vector construction

The Gateway™ destination vector, pHAtC binary vector (Kim et al. 2016), was used to construct OsU6a::pHAtC carrying the OsU6a promoter to express sgRNA. A 472-bp DNA fragment containing the OsU6a promoter (Ma et al. 2015c) was amplified from the genomic DNA of Kitaake using primers, EcoRI_OsU6a_F (5′-GGAATTCTTTTTTCCTGTAGTTTTCCCAC-3′) and XhoI_OsU6a_R (5′-GCTCGAGACACCTGCCTCCAATCCGGCAGCCAAGCCAGCACCC-3′). The PCR product was cloned into the pGEM®-T Easy Vector according to the manufacturer’s instructions (Promega, USA), and the insert was confirmed by Sanger sequencing. The OsU6a promoter was cut out from the pGEM®-T Easy Vector using *EcoR*I + *Xho*I and cloned into the pHAtC, generating an OsU6a::pHAtC vector.

### Cloning of sgRNA expression vector

The OsU6a::*xa13m-sgRNA*/pHAtC vector expressing sgRNA for *xa13/Os8N3* (*xa13m-sgRNA*) was constructed according to the method previously described (Kim et al. 2016). Briefly, the target sequence (*xa13m*) for *Os8N3* editing of Kitaake was designed by the CRISPR-RGEN Tools website (http://rgenome.ibs.re.kr) (Park et al. 2015). The sgRNA templates (*xa13m*) for *Os8N3* were annealed using two primers, 5′-GATTGCTTGTCCATGGCTAACCCGG-3′ and 5′- AAACCCGGGTTAGCCATGGACAAGC-3′, and cloned into *Aar*I-digested OsU6a::pHAtC. Construction of the sgRNA expression vector, OsU6a::*xa13m-sgRNA*/pHAtC, and its flanking sequences were confirmed by Sanger sequencing.

### Rice transformations

Rice transformations were carried out as previously described (Chern et al. 2005). *Agrobacterium tumefaciens* strain LBA4404 was used to infect callus tissue induced from Kitaake seeds. Transformants carrying OsU6a::*xa13m-sgRNA*/pHAtC constructs were selected using hygromycin. Transgenic Kitaake plants overexpressing *xa13m-sgRNA* (OsU6a *xa13m*/Kit) were confirmed by PCR using *Cas9*-specific primers, Cas9_RT_F (5′-CGAGCTGACCAAGGTGAAGT-3′) and Cas9_RT_R (5′-CGTTGATAAGCTTGCGGCTC-3′).

### Expression

For reverse transcription polymerase chain reaction (RT-PCR) analysis of *Cas9* and *sgxa13* transgenes, total RNA was extracted from fully expanded leaves of OsU6a *xa13m*/Kit plants using TRIzol reagent (Invitrogen, USA). First-strand cDNA was synthesized using quantified RNA (5 μg of total RNA). Expression of Cas9 was confirmed by RT-PCR using Cas9_RT_F and Cas9_RT_R. Meanwhile, the *rEFla* cDNA fragment was amplified as a control using specific primers, rEF1a1048F (5′-ACTGCCACACCTCCCACATTG-3′) and rEF1a1552R (5′-CAACAGTCGAAGGGCAATAATAAGTC-3′).

### Identification of mutant transgenic plants

Rice genomic DNA was extracted from Kitaake leaves and transgenic OsU6a *xa13m*/Kit plants. All transgenic hygromycin-resistant T_0_ plants were analyzed by PCR using the Cas9-specific primers, Cas9_RT_F and Cas9_RT_R. Subsequently, the DNA fragment across the *xa13* target site was amplified from the genomic DNAs of all PCR-positive plants using *xa13*-specific primers, xa13_cas9 60-79_F (5′-TCTGGCTAGTTTCTAGCTGG-3′) and xa13_cas9 nuclease_R (5′-TGCATGAGCTGAAGCTAGGG-3′). The PCR amplicons were then directly sequenced using primer xa13_cas9 60-79_F. The sequencing chromatograms with superimposed peaks of bi-allelic and heterozygous mutations were decoded using the Degenerate Sequence Decoding method (http://skl.scau.edu.cn/dsdecode/) (Liu et al. 2015; Ma et al. 2015a).

### *Xoo* inoculation and determination of bacterial populations

For *Xoo* inoculation, Kitaake, XA21, and transgenic OsU6a *xa13m*/Kit plants were grown in a greenhouse normally until they reached the eight-week stage, unless otherwise stated. PXO99 was used to inoculate rice plants using the scissors dip method (Song et al. 1995; Chern et al. 2005). For lesion length measurements, at least three inoculated leaves were measured to calculate the average and standard deviation 12 days after inoculation (DAI). Representative leaves were photographed 12 DAI. For *Xoo* colony counts from inoculated leaves 0 and 12 DAI, 20 cm of leaf tissue from the top, including lesions and tissue showing no lesions, was ground up and resuspended in 10 ml water to harvest bacteria. The extract was diluted accordingly and plated out on PSA plates containing 15.0 mg/L cephalexin. Plates were incubated at 28 °C for two days, and then colony forming units (CFU) were counted. Statistical analysis was performed using Tukey’s HSD test.

### Pollen viability tests

Pollen viability was evaluated as previously described (Chhun et al. 2007). Before flowering, six anthers from Kitaake and transgenic OsU6a *xa13m*/Kit plants were removed and crushed into a fine powder. Pollen grains were stained with 10 μl I_2_-KI solution (1% I_2_, 3% KI) and 1 μl of stained pollen grains was harvested to observe fertile and infertile pollen under a light microscope. Dark-stained pollen grains were considered viable and the percentage of pollen viability was calculated relative to the total pollen counted in five microscopic images. Seed viability represents the percentage of spikelets that set seed per total number. Statistical analysis was performed using Tukey’s HSD test.

## Additional files

Additional file 1: **Figure S1.** Sequence comparison of OsU6a promoters from Japonica cultivar Kitaake and Indica cultivar 93–11. (PDF 72 kb)

Additional file 2: **Figure S2.** Sequencing chromatogram at the target site of *Os8N3* in the CRISPR/Cas9-induced plants (OsU6a *xa13m*/Kit T_0_). The vertical arrowhead indicates an expected cleavage site. (PDF 105 kb)

Additional file 3: **Figure S3.** Sequencing chromatogram at the target site of *Os8N3* in the CRISPR/Cas9-induced plants (OsU6a *xa13m*/Kit T_1_). The vertical arrowhead indicates an expected cleavage site. (PDF 232 kb)

Additional file 4: **Figure S4.** Sequencing chromatogram at the target site of *Os8N3* in the CRISPR/Cas9-induced plants (OsU6a *xa13m*/Kit T_2_). The vertical arrowhead indicates an expected cleavage site. (PDF 188 kb)

Additional file 5: **Figure S5.** Sequencing chromatogram at the target site of *Os8N3* in the CRISPR/Cas9-induced plants (OsU6a *xa13m*/Kit T_3_). The vertical arrowhead indicates an expected cleavage site. (PDF 196 kb)

Additional file 6 **Figure S6.** Gross morphology of Kitaake and two homozygous *Os8N3* mutant lines, T_3_ progeny of 3A-6-1 and 4A-1-7. (PDF 72 kb)

Additional file 7: **Figure S7.** Seed-setting rates of homozygous *xa13* mutants. a Representative panicles from Kitaake, homozygous mutant (T_3_, 3A-6-1-2), and homozygous mutant (T_3_, 4A-1-7-4). b Seed-setting rates of Kitaake, homozygous mutant (progeny of 3A-6-1), and homozygous mutant (progeny of 4A-1-7). c Mature caryopses of Kitaake, homozygous mutant (T_3_, 3A-6-1-1), and homozygous mutant (T_3_, 4A-1-7-1). Scale bars, 2.5 mm. (PDF 78 kb)

## Data Availability

All data generated or analysed during this study are included in this published article and its supplementary information files.

## Abbreviations

CRISPR/Cas9: Clustered regularly interspaced short palindromic repeats/CRISPR-associated protein-9 nuclease
EBE: Effector binding element
TAL effector: Transcription activator-like effector
*Xoo* PR6: *Xanthomonas oryzae* pv. *oryzae* Philippine Race 6
