# Correction to: CRISPR/Cas9-targeted mutagenesis of Os8N3 in rice to confer resistance to *Xanthomonas oryzae pv. Oryzae*

**DOI:** 10.1186/s12284-019-0331-9

**Published:** 2019-09-13

**Authors:** Young-Ah Kim, Hyeran Moon, Chang-Jin Park

**Affiliations:** 1 Department of Plant Biotechnology, Sejong University, Seoul, 05006 South Korea; 2 Department of Molecular Biology, Sejong University, Seoul, 05006 South Korea; 3 Plant Engineering Research Institute, Sejong University, Seoul, 05006 South Korea

**Correction to: Rice 12:67**

**Fig. 3:**
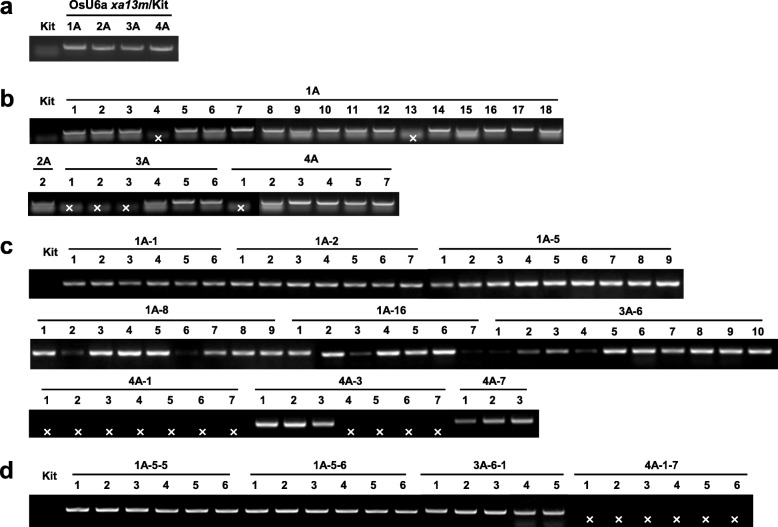
Generation of transgenic rice plants carrying the Cas9 transgene with a sgRNA targeting the Os8N3 gene. Genotyping was performed using the specific primers for Cas9, Cas9_RT_F and Cas9_RT_R (see Fig. 2b), from four independently transformed plants and their progenies (OsU6a xa13m/Kit T_0_, T_1_, T_2_, and T_3_ generations). Genomic DNAs were extracted from Kit (Kitaake) and OsU6a xa13m/Kit T0 (**a**), T_1_ (_b_), T_2_ (_c_), and T_3_ (**d**). ‘ × ’ indicates PCR negative

**https://doi.org/10.1186/s12284-019-0325-7**

It was highlighted that in the original article (Kim, 2019) all PCR bands in Fig. 3a did not appear. This Correction article shows the correct Fig. 3. The original article has been updated.
