# Supplementary material for: CRISPR/Cas9-targeted mutagenesis of Os8N3 in rice to confer resistance to Xanthomonas oryzae pv. oryzae
Source: Rice (N Y). 2019 Aug 24;12:67. doi: 10.1186/s12284-019-0325-7 (PMC6708514; doi:10.1186/s12284-019-0325-7)
Supplement: Supplementary file 1 — Figure S1. Sequence comparison of OsU6a promoters from Japonica cultivar Kitaake and Indica cultivar 93–11. (PDF 72 kb) [file 12284_2019_325_MOESM1_ESM.pdf]

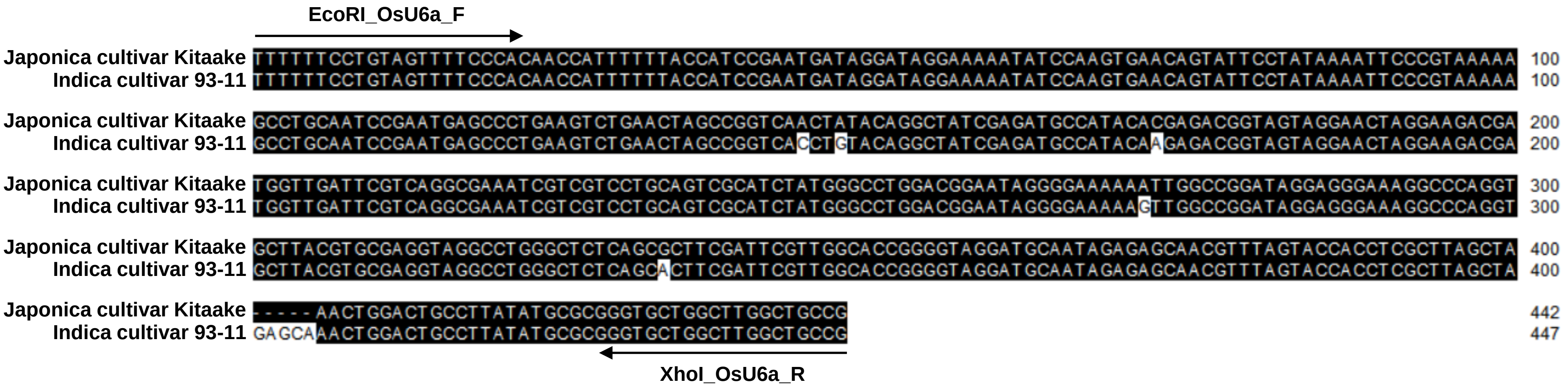
EcoRI_OsU6a_F
Japonica cultivar Kitaake TTTTTTCCTGTAGTTTTCCCACAACCATTTTTTACCATCCGAATGATAGGATAGGAAAAATATCCAAGTGAACAGTATTCCTATAAAATTCCCGTAAAAA 100
Indica cultivar 93-11 TTTTTTCCTGTAGTTTTCCCACAACCATTTTTTACCATCCGAATGATAGGATAGGAAAAATATCCAAGTGAACAGTATTCCTATAAAATTCCCGTAAAAA 100
Japonica cultivar Kitaake GCCTGCAATCCGAATGAGCCCTGAAGTCTGAACTAGCCGGTCAACTATACAGGCTATCGAGATGCCATACACGAGACGGTAGTAGGAACTAGGAAGACGA 200
Indica cultivar 93-11 GCCTGCAATCCGAATGAGCCCTGAAGTCTGAACTAGCCGGTCACCTGTACAGGCTATCGAGATGCCATACAAGAGACGGTAGTAGGAACTAGGAAGACGA 200
Japonica cultivar Kitaake TGGTTGATTCGTCAGGCGAAATCGTCGTCCTGCAGTCGCATCTATGGGCCTGGACGGAATAGGGGAAAAAATTGGCCGGATAGGAGGGAAAGGCCCAGGT 300
Indica cultivar 93-11 TGGTTGATTCGTCAGGCGAAATCGTCGTCCTGCAGTCGCATCTATGGGCCTGGACGGAATAGGGGAAAAAGTTGGCCGGATAGGAGGGAAAGGCCCAGGT 300
Japonica cultivar Kitaake GCTTACGTGCGAGGTAGGCCTGGGCTCTCAGCGCTTCGATTCGTTGGCACCGGGGTAGGATGCAATAGAGAGCAACGTTTAGTACCACCTCGCTTAGCTA 400
Indica cultivar 93-11 GCTTACGTGCGAGGTAGGCCTGGGCTCTCAGCACTTCGATTCGTTGGCACCGGGGTAGGATGCAATAGAGAGCAACGTTTAGTACCACCTCGCTTAGCTA 400
Japonica cultivar Kitaake -----AACTGGACTGCCTTATATGCGCGGGTGCTGGCTTGGCTGCCG 442
Indica cultivar 93-11 GAGCAAACTGGACTGCCTTATATGCGCGGGTGCTGGCTTGGCTGCCG 447
XhoI_OsU6a_R
