# Supplementary material for: CRISPR/Cas9-targeted mutagenesis of Os8N3 in rice to confer resistance to Xanthomonas oryzae pv. oryzae
Source: Rice (N Y). 2019 Aug 24;12:67. doi: 10.1186/s12284-019-0325-7 (PMC6708514; doi:10.1186/s12284-019-0325-7)
Supplement: Supplementary file 2 — Figure S2. Sequencing chromatogram at the target site of Os8N3 in the CRISPR/Cas9-induced plants (OsU6a xa13m/Kit T0). The vertical arrowhead indicates an expected cleavage site. (PDF 105 kb) [file 12284_2019_325_MOESM2_ESM.pdf]

**Kitaake**

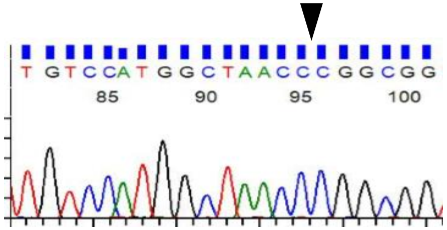


WT: TGTCCATGGCTAACC-CGGCGG
WT: TGTCCATGGCTAACC-CGGCGG

**Wild-type**

**OsU6a *xa13m*/Kit $T_0$**

**1A**

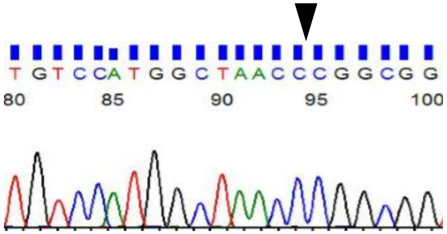


WT: TGTCCATGGCTAACC-CGGCGG
WT: TGTCCATGGCTAACC-CGGCGG

**Wild-type**

**2A**

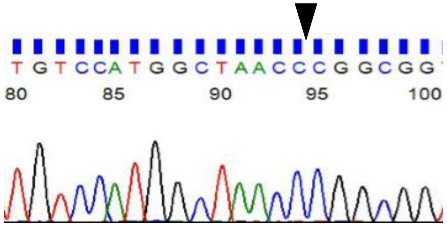


WT: TGTCCATGGCTAACC-CGGCGG
WT: TGTCCATGGCTAACC-CGGCGG

**Wild-type**

**3A**

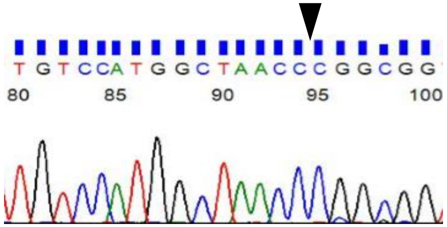


WT: TGTCCATGGCTAACC-CGGCGG
WT: TGTCCATGGCTAACC-CGGCGG

**Wild-type**

**4A**

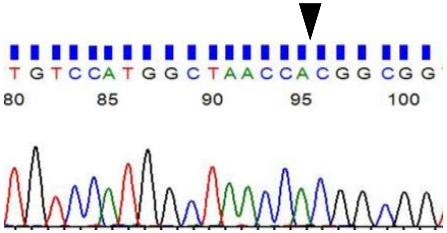


M1: TGTCCATGGCTAACCACGGCGG
M1: TGTCCATGGCTAACCACGGCGG

**Homozygote**
