# Supplementary material for: CRISPR/Cas9-targeted mutagenesis of Os8N3 in rice to confer resistance to Xanthomonas oryzae pv. oryzae
Source: Rice (N Y). 2019 Aug 24;12:67. doi: 10.1186/s12284-019-0325-7 (PMC6708514; doi:10.1186/s12284-019-0325-7)
Supplement: Supplementary file 3 — Figure S3. Sequencing chromatogram at the target site of Os8N3 in the CRISPR/Cas9-induced plants (OsU6a xa13m/Kit T1). The vertical arrowhead indicates an expected cleavage site. (PDF 232 kb) [file 12284_2019_325_MOESM3_ESM.pdf]

	Plant	Chromatogram	Sequence	Genotype
Kitaake		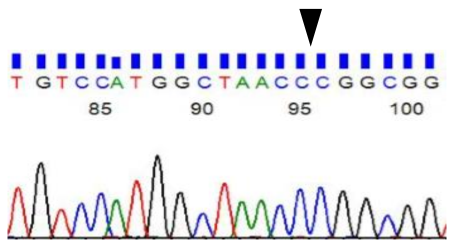	WT: TGTCCATGGCTAACC-CGGCGG<br>WT: TGTCCATGGCTAACC-CGGCGG	**Wild-type**
OsU6a *xa13m*/Kit T_1	**1A-1**	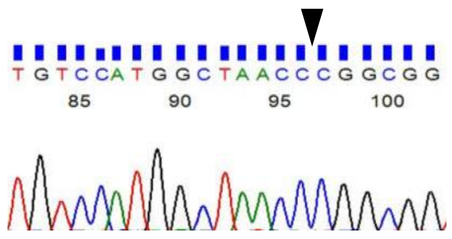	WT: TGTCCATGGCTAACC-CGGCGG<br>WT: TGTCCATGGCTAACC-CGGCGG	**Wild-type**
	**1A-5**	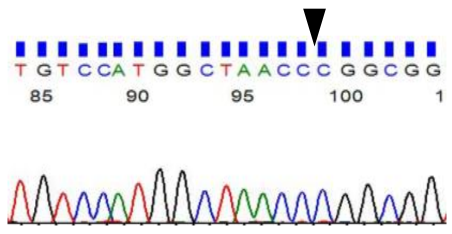	WT: TGTCCATGGCTAACC-CGGCGG<br>WT: TGTCCATGGCTAACC-CGGCGG	**Wild-type**
	**1A-8**	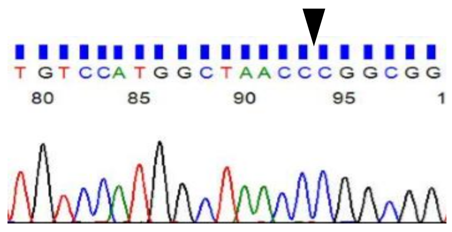	WT: TGTCCATGGCTAACC-CGGCGG<br>WT: TGTCCATGGCTAACC-CGGCGG	**Wild-type**
	**1A-16**	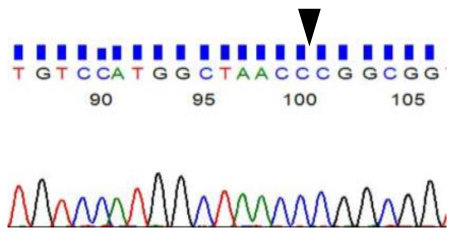	WT: TGTCCATGGCTAACC-CGGCGG<br>WT: TGTCCATGGCTAACC-CGGCGG	**Wild-type**
	**2A-2**	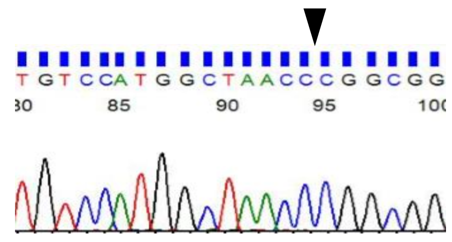	WT: TGTCCATGGCTAACC-CGGCGG<br>WT: TGTCCATGGCTAACC-CGGCGG	**Wild-type**
	**3A-2**	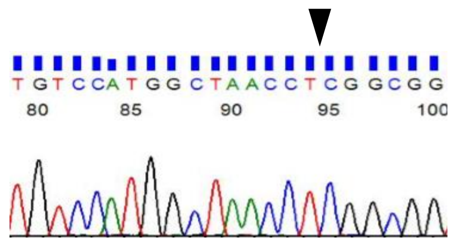	M2: TGTCCATGGCTAACCTCGGCGG<br>M2: TGTCCATGGCTAACCTCGGCGG	**Homozygote**
	**3A-3**	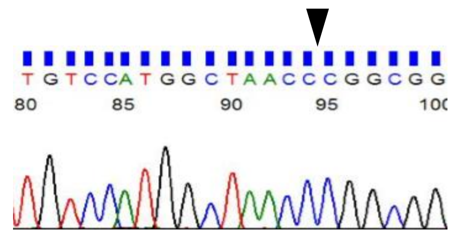	WT: TGTCCATGGCTAACC-CGGCGG<br>WT: TGTCCATGGCTAACC-CGGCGG	**Wild-type**
	**3A-4**	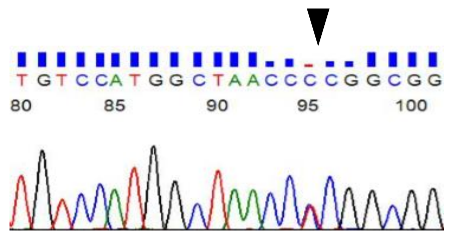	M3: TGTCCATGGCTAACCCCGGCGG<br>M2: TGTCCATGGCTAACCTCGGCGG	**Bi-allele**
	**3A-5**	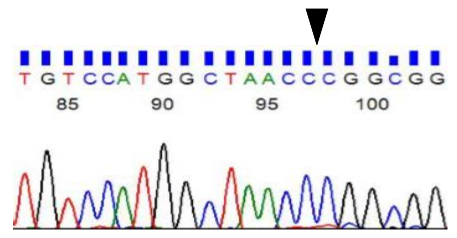	WT: TGTCCATGGCTAACC-CGGCGG<br>WT: TGTCCATGGCTAACC-CGGCGG	**Wild-type**
	**3A-6**	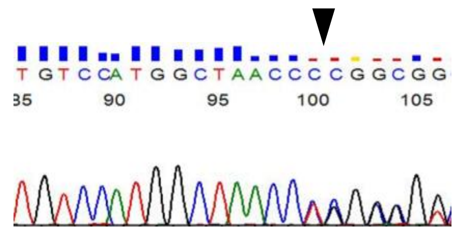	WT: TGTCCATGGCTAACC-CGGCGG<br>M2: TGTCCATGGCTAACCTCGGCGG	**Heterozygote**
	**4A-1**	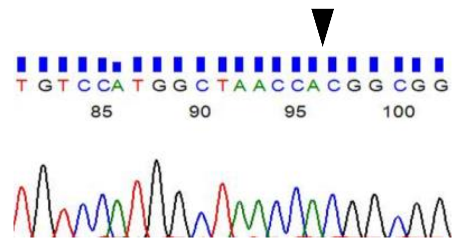	M1: TGTCCATGGCTAACCACGGCGG<br>M1: TGTCCATGGCTAACCACGGCGG	**Homozygote**
	**4A-2**	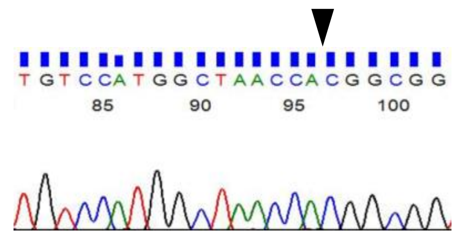	M1: TGTCCATGGCTAACCACGGCGG<br>M1: TGTCCATGGCTAACCACGGCGG	**Homozygote**
	**4A-3**	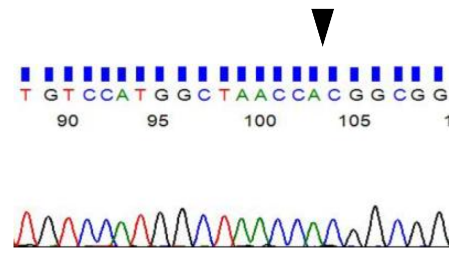	M1: TGTCCATGGCTAACCACGGCGG<br>M1: TGTCCATGGCTAACCACGGCGG	**Homozygote**
