# Supplementary material for: CRISPR/Cas9-targeted mutagenesis of Os8N3 in rice to confer resistance to Xanthomonas oryzae pv. oryzae
Source: Rice (N Y). 2019 Aug 24;12:67. doi: 10.1186/s12284-019-0325-7 (PMC6708514; doi:10.1186/s12284-019-0325-7)
Supplement: Supplementary file 4 — Figure S4. Sequencing chromatogram at the target site of Os8N3 in the CRISPR/Cas9-induced plants (OsU6a xa13m/Kit T2). The vertical arrowhead indicates an expected cleavage site. (PDF 188 kb) [file 12284_2019_325_MOESM4_ESM.pdf]

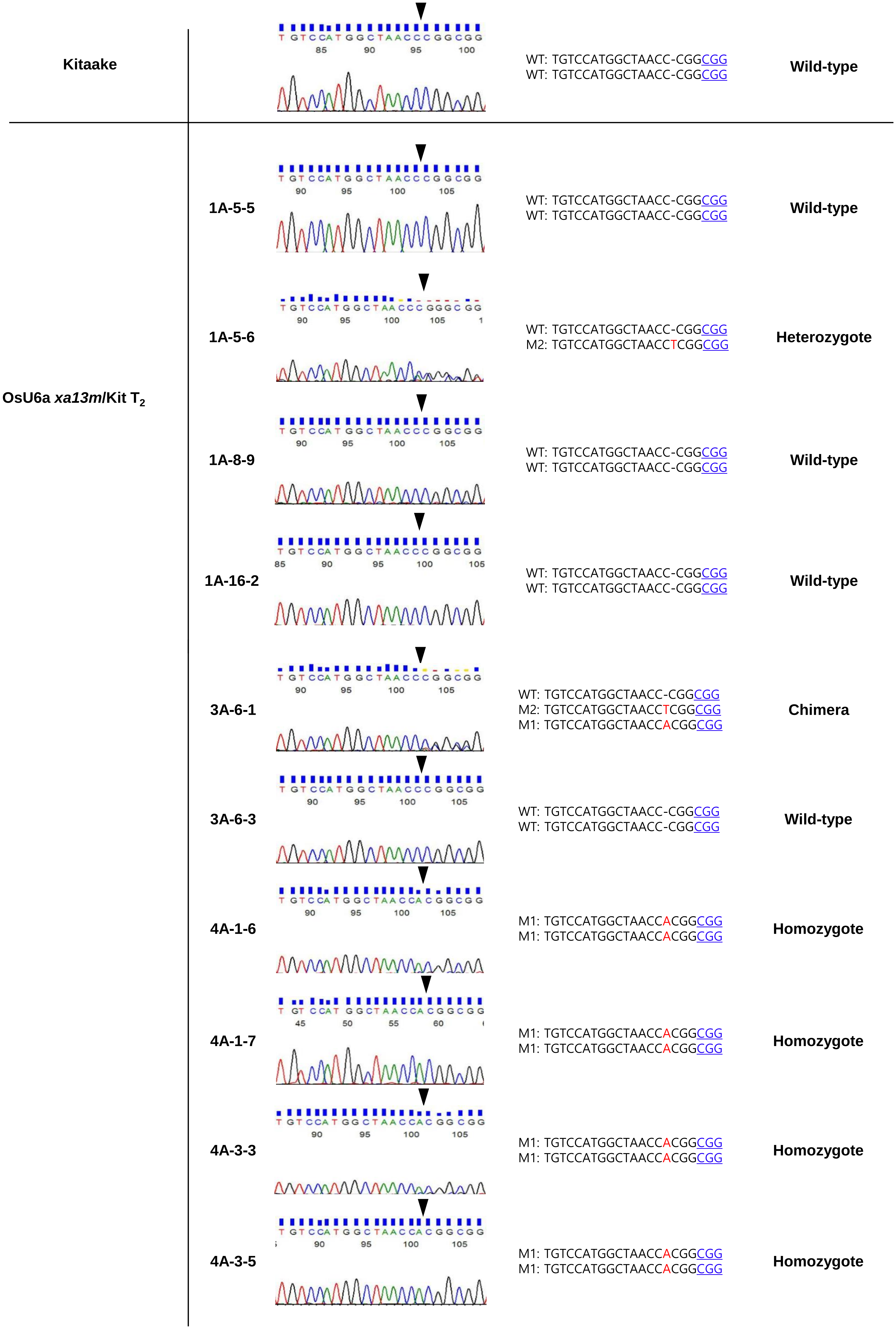
Kitaake
WT: TGTCCATGGCTAACC-CGGCGG
WT: TGTCCATGGCTAACC-CGGCGG
Wild-type
OsU6a xa13m/Kit T2
1A-5-5
WT: TGTCCATGGCTAACC-CGGCGG
WT: TGTCCATGGCTAACC-CGGCGG
Wild-type
1A-5-6
WT: TGTCCATGGCTAACC-CGGCGG
M2: TGTCCATGGCTAACCTCGGCGG
Heterozygote
1A-8-9
WT: TGTCCATGGCTAACC-CGGCGG
WT: TGTCCATGGCTAACC-CGGCGG
Wild-type
1A-16-2
WT: TGTCCATGGCTAACC-CGGCGG
WT: TGTCCATGGCTAACC-CGGCGG
Wild-type
3A-6-1
WT: TGTCCATGGCTAACC-CGGCGG
M2: TGTCCATGGCTAACCTCGGCGG
M1: TGTCCATGGCTAACCACGGCGG
Chimera
3A-6-3
WT: TGTCCATGGCTAACC-CGGCGG
WT: TGTCCATGGCTAACC-CGGCGG
Wild-type
4A-1-6
M1: TGTCCATGGCTAACCACGGCGG
M1: TGTCCATGGCTAACCACGGCGG
Homozygote
4A-1-7
M1: TGTCCATGGCTAACCACGGCGG
M1: TGTCCATGGCTAACCACGGCGG
Homozygote
4A-3-3
M1: TGTCCATGGCTAACCACGGCGG
M1: TGTCCATGGCTAACCACGGCGG
Homozygote
4A-3-5
M1: TGTCCATGGCTAACCACGGCGG
M1: TGTCCATGGCTAACCACGGCGG
Homozygote
