# Supplementary material for: CRISPR/Cas9-targeted mutagenesis of Os8N3 in rice to confer resistance to Xanthomonas oryzae pv. oryzae
Source: Rice (N Y). 2019 Aug 24;12:67. doi: 10.1186/s12284-019-0325-7 (PMC6708514; doi:10.1186/s12284-019-0325-7)
Supplement: Supplementary file 5 — Figure S5. Sequencing chromatogram at the target site of Os8N3 in the CRISPR/Cas9-induced plants (OsU6a xa13m/Kit T3). The vertical arrowhead indicates an expected cleavage site. (PDF 196 kb) [file 12284_2019_325_MOESM5_ESM.pdf]

**Kitaake**		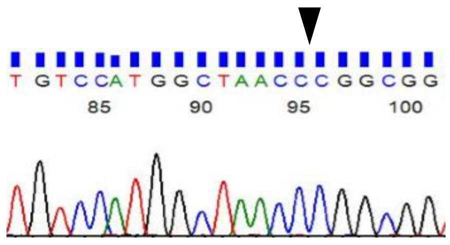	WT: TGTCCATGGCTAACC-CGGCGG<br>WT: TGTCCATGGCTAACC-CGGCGG	**Wild-type**
**OsU6a *xa13m*/Kit T_3**	**1A-5-5-1**	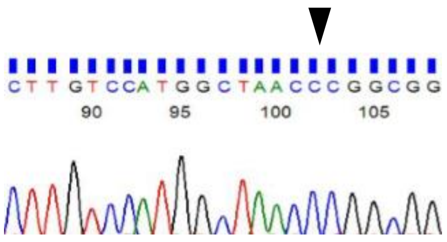	WT: TGTCCATGGCTAACC-CGGCGG<br>WT: TGTCCATGGCTAACC-CGGCGG	**Wild-type**
	**1A-5-6-3**	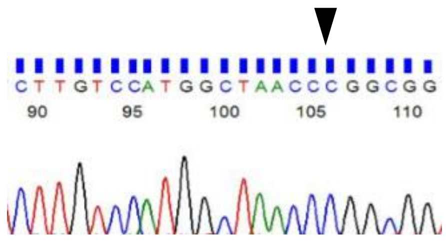	WT: TGTCCATGGCTAACC-CGGCGG<br>M2: TGTCCATGGCTAACC-CGGCGG	**Wild-type**
	**1A-5-6-4**	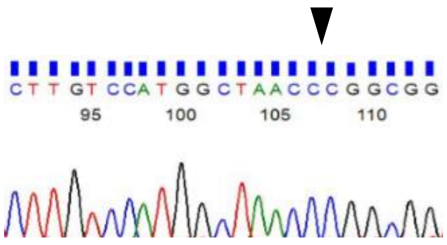	WT: TGTCCATGGCTAACC-CGGCGG<br>WT: TGTCCATGGCTAACC-CGGCGG	**Wild-type**
	**1A-5-6-5**	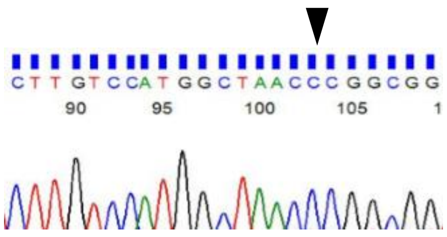	WT: TGTCCATGGCTAACC-CGGCGG<br>WT: TGTCCATGGCTAACC-CGGCGG	**Wild-type**
	**1A-5-6-6**	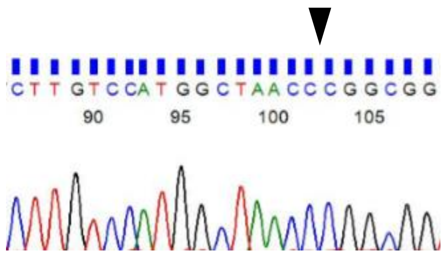	WT: TGTCCATGGCTAACC-CGGCGG<br>WT: TGTCCATGGCTAACC-CGGCGG	**Wild-type**
	**3A-6-1-1**	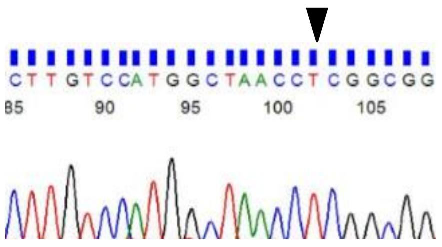	M1: TGTCCATGGCTAACCTCGGCGG<br>M1: TGTCCATGGCTAACCTCGGCGG	**Homozygote**
	**3A-6-1-2**	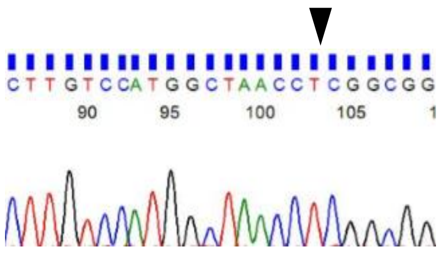	M1: TGTCCATGGCTAACCTCGGCGG<br>M1: TGTCCATGGCTAACCTCGGCGG	**Homozygote**
	**3A-6-1-3**	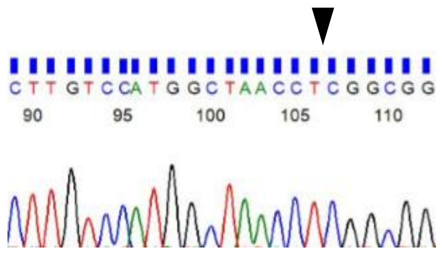	M1: TGTCCATGGCTAACCTCGGCGG<br>M1: TGTCCATGGCTAACCTCGGCGG	**Homozygote**
	**3A-6-1-4**	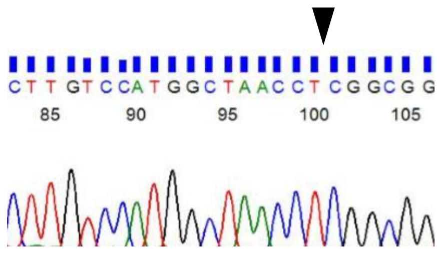	M1: TGTCCATGGCTAACCTCGGCGG<br>M1: TGTCCATGGCTAACCTCGGCGG	**Homozygote**
	**3A-6-1-5**	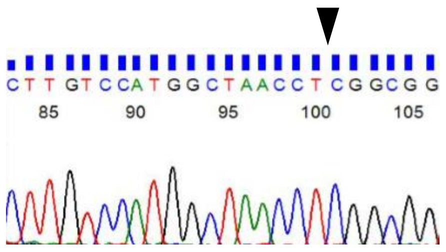	M1: TGTCCATGGCTAACCTCGGCGG<br>M1: TGTCCATGGCTAACCTCGGCGG	**Homozygote**
	**4A-1-7-1**	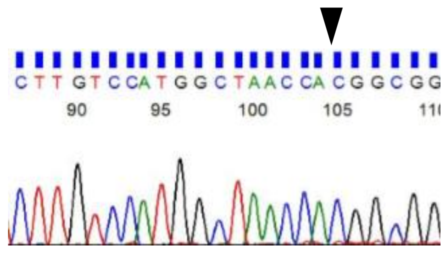	M2: TGTCCATGGCTAACCACGGCGG<br>M2: TGTCCATGGCTAACCACGGCGG	**Homozygote**
	**4A-1-7-6**	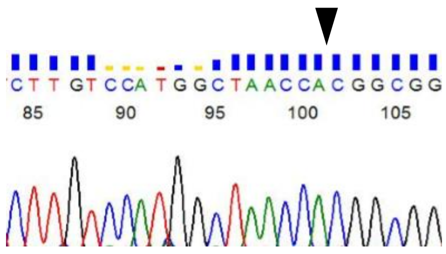	M2: TGTCCATGGCTAACCACGGCGG<br>M2: TGTCCATGGCTAACCACGGCGG	**Homozygote**
