# Supplementary figures and images for: CRISPR/Cas9-targeted mutagenesis of Os8N3 in rice to confer resistance to Xanthomonas oryzae pv. oryzae
Source: Rice (N Y). 2019 Aug 24;12:67. doi: 10.1186/s12284-019-0325-7 (PMC6708514; doi:10.1186/s12284-019-0325-7)

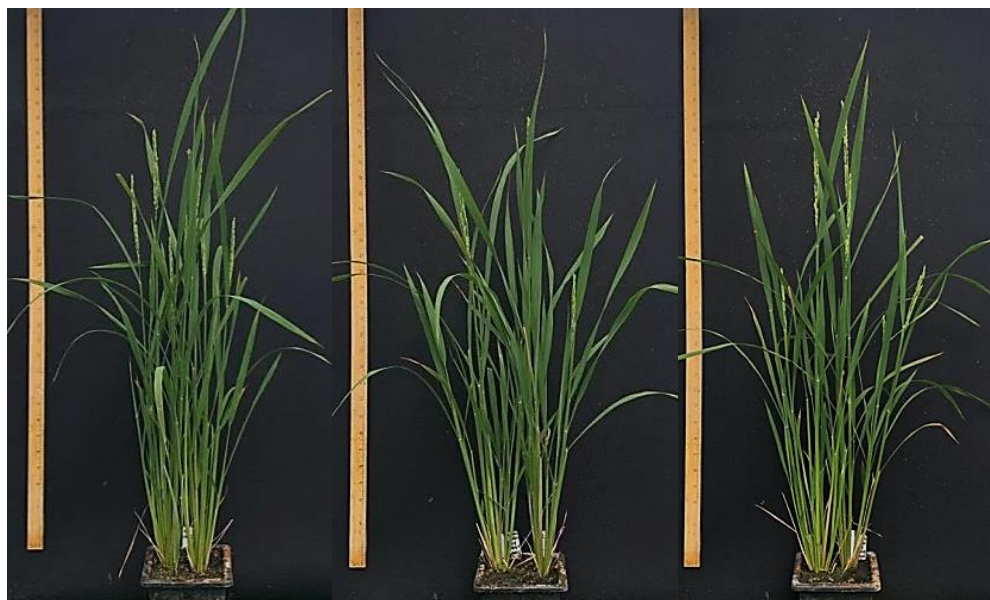

**Kitaake**

**3A-6-1-4  
3A-6-1-5**

**4A-1-7-6  
4A-1-7-7**

---

**OsU6a *xa13m*/Kit T<sub>3</sub>**

Supplement: Supplementary file 6 — Figure S6. Gross morphology of Kitaake and two homozygous Os8N3 mutant lines, T3 progeny of 3A-6-1 and 4A-1-7. (PDF 72 kb) [file 12284_2019_325_MOESM6_ESM.pdf]

**a**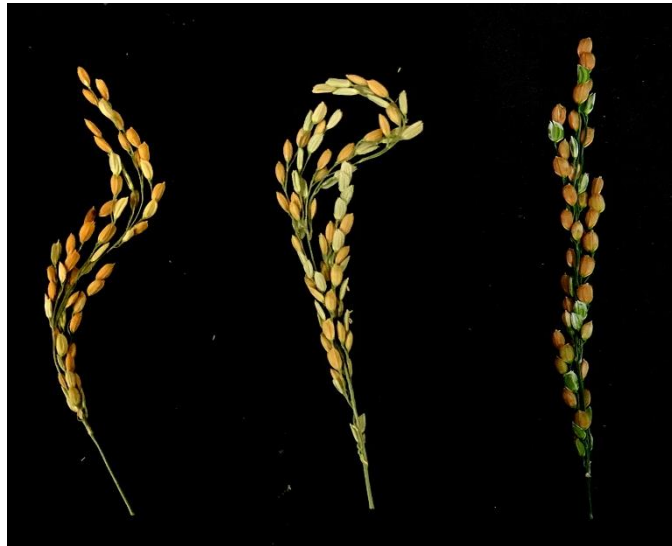

Kitaake

3A-6-1-2

4A-1-7-4

OsU6a *xa13m*/Kit**b**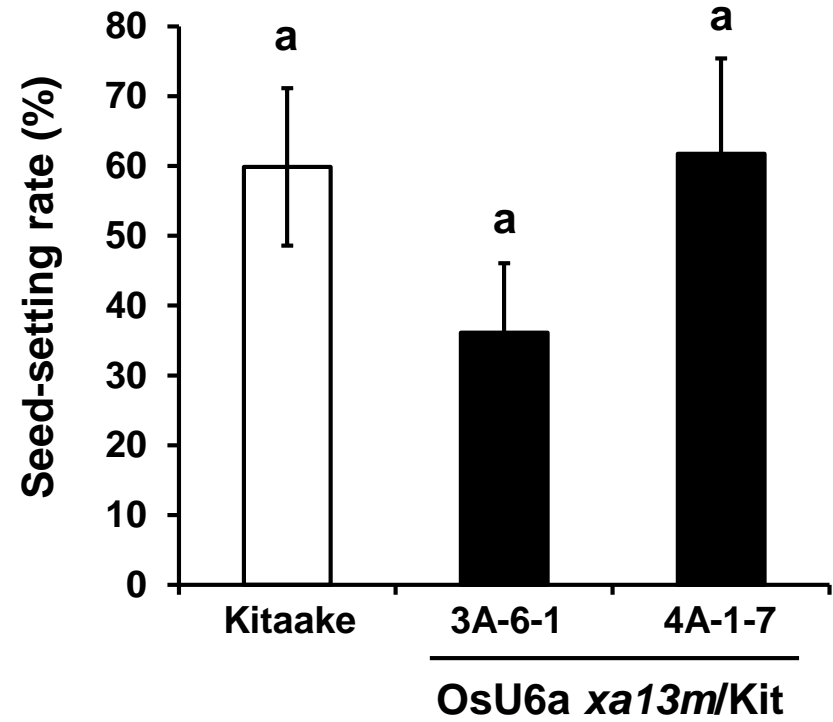**c**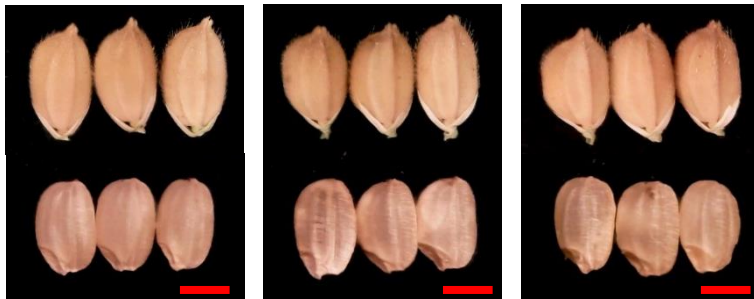

Kitaake

3A-6-1-1

4A-1-7-1

OsU6a *xa13m*/Kit

Supplement: Supplementary file 7 — Figure S7. Seed-setting rates of homozygous xa13 mutants. a Representative panicles from Kitaake, homozygous mutant (T3, 3A-6-1-2), and homozygous mutant (T3, 4A-1-7-4). b Seed-setting rates of Kitaake, homozygous mutant (progeny of 3A-6-1), and homozygous mutant (progeny of 4A-1-7). c Mature caryopses of Kitaake, homozygous mutant (T3, 3A-6-1-1), and homozygous mutant (T3, 4A-1-7-1). Scale bars, 2.5 mm. (PDF 78 kb) [file 12284_2019_325_MOESM7_ESM.pdf]
